# Metabolism and Innate Immunity Meet at the Mitochondria

**DOI:** 10.3389/fcell.2021.720490

**Published:** 2021-07-27

**Authors:** Amir Bahat, Thomas MacVicar, Thomas Langer

**Affiliations:** ^1^Max Planck Institute for Biology of Ageing, Cologne, Germany; ^2^Cologne Excellence Cluster on Cellular Stress Responses in Aging-Associated Diseases (CECAD), University of Cologne, Cologne, Germany

**Keywords:** mitochondria, metabolism, innate immunity, mitochondrial DNA, MAVS, CGAS, STING

## Abstract

Mitochondria are master regulators of metabolism and have emerged as key signalling organelles of the innate immune system. Each mitochondrion harbours potent agonists of inflammation, including mitochondrial DNA (mtDNA), which are normally shielded from the rest of the cell and extracellular environment and therefore do not elicit detrimental inflammatory cascades. Mitochondrial damage and dysfunction can lead to the cytosolic and extracellular exposure of mtDNA, which triggers inflammation in a number of diseases including autoimmune neurodegenerative disorders. However, recent research has revealed that the extra-mitochondrial exposure of mtDNA is not solely a negative consequence of mitochondrial damage and pointed to an active role of mitochondria in innate immunity. Metabolic cues including nucleotide imbalance can stimulate the release of mtDNA from mitochondria in order to drive a type I interferon response. Moreover, important effectors of the innate immune response to pathogen infection, such as the mitochondrial antiviral signalling protein (MAVS), are located at the mitochondrial surface and modulated by the cellular metabolic status and mitochondrial dynamics. In this review, we explore how and why metabolism and innate immunity converge at the mitochondria and describe how mitochondria orchestrate innate immune signalling pathways in different metabolic scenarios. Understanding how cellular metabolism and metabolic programming of mitochondria are translated into innate immune responses bears relevance to a broad range of human diseases including cancer.

## Introduction

Mitochondria are dynamic double-membrane organelles responsible for ATP production, the biosynthesis of macromolecules including lipids, proteins, and nucleotides and for cellular redox status. They serve as metabolic hubs and respond to intrinsic cues and environmental stressors with an incredible degree of plasticity, which enables them to participate in key signalling pathways such as programmed cell death and redox homoeostasis (Bahat and Gross, 2019; Chakrabarty and Chandel, 2021). Apoptotic signals trigger the oligomerisation of pro-apoptotic effector proteins BAX and BAK on the mitochondrial surface, leading to mitochondrial outer membrane (OM) permeabilisation and the release of pro-apoptotic signals into the cytosol to activate caspases and trigger cell death (Flores-Romero et al., 2020). Mitochondria thus play a central role in programmed cell death and this places them as important regulators of cellular responses to pathogen infection. Mitochondrial cell death pathways are directly and indirectly manipulated by viruses to combat innate immune responses and promote infection (Imre, 2020). However, the interplay between mitochondria and innate immunity goes well beyond the control of host cell death or survival. Recent work identified numerous innate immune responses that are controlled by mitochondria and revealed reciprocal relationships that exist between innate immunity and cellular metabolism.

The innate immune system is poised on the front line of defence against infection. Invading pathogens are recognised by a number of pattern recognition receptors (PRRs) that have evolved to sense a wide array of pathogen-associated molecular patterns (PAMPs) such as bacterial lipopolysaccharide (LPS) and viral nucleic acids (Brubaker et al., 2015). The majority of PRRs belong to four protein families: Toll-like receptors (TLRs), C-type lectin receptors (CLRs), NOD-like receptors (NLRs), and retinoic acid-inducible gene-I (RIG-I)-like receptors (RLRs). PRR activation triggers a variety of innate immune responses such as the type I interferon (IFN) pathway and expression of pro-inflammatory cytokines and interferon stimulated genes (ISGs), in order to concoct an anti-microbial environment and prime the adaptive immune system (Brubaker et al., 2015). A well described way by which mitochondria support the transduction of innate immune signals occurs downstream of RLR activation during RNA virus infection (Rehwinkel and Gack, 2020). Detection of cytosolic dsRNA by the RLR proteins, RIG-I, or melanoma differentiation-associated gene 5 (MDA5), causes a conformational change in these proteins, which leads them to the RLR adaptor protein called mitochondrial antiviral signalling protein (MAVS) (Kawai et al., 2005; Meylan et al., 2005; Seth et al., 2005; Xu et al., 2005). RIG-I and MDA5 utilise homotypic caspase activation and recruitment domain (CARD)-binding to interact with MAVS, which is anchored on the OM surface (Seth et al., 2005) as well as mitochondrial associated membranes (Horner et al., 2011) and peroxisomes (Dixit et al., 2010). This interaction leads to MAVS aggregation and subsequent activation of NF-kB and interferon regulatory factor (IRF) signalling pathways (Hou et al., 2011).

Innate immune responses are also triggered in the absence of pathogen infection, commonly referred to as sterile inflammation, whereby PRRs and non-PRR receptors sense damage-associated molecular patterns (DAMPs) that originate from within the cell itself (Gong et al., 2020). Besides harbouring pro-apoptotic signals, mitochondria contain numerous potent immunostimulatory DAMPs, including their own genome, that engage the innate immune system upon exposure to the cytosol or release into the extracellular environment. Mitochondria house circular molecules of mitochondrial DNA (mtDNA) within the matrix, which encode 13 subunits of the respiratory chain, 22 transfer RNAs (tRNA), and 2 ribosomal RNAs (rRNA). The exposure of mtDNA has long been known to be immunostimulatory likely owing to its bacterial origin and the release of mtDNA into the cytosol can activate a number of PRRs to trigger a variety of innate immune responses (Collins et al., 2004).

The DNA sensor cyclic GMP-AMP (cGAMP) synthase (cGAS) is one such PRR and binds cytosolic double-stranded DNA (dsDNA) derived from micronuclei, mitochondria, or invading pathogens. dsDNA binding to cGAS leads to its enzymatic activation and the generation of the second messenger cGAMP. cGAMP activates stimulator of interferon genes (STING) at the endoplasmic reticulum (ER), resulting in the recruitment of the tank binding kinase 1 (TBK1) and activation of the IFN signalling pathway (Rongvaux et al., 2014; White et al., 2014; West et al., 2015). While the release of mtDNA can support the antiviral response orchestrated by PRRs, it can also cause chronic inflammation in disease. Notably, exposed mtDNA is also recognised by endosomal TLR9 (Zhang et al., 2010; Shepard, 2020) or, if oxidised, can stimulate the NOD-like receptor family pyrin domain containing 3 (NLRP3) inflammasome (Nakahira et al., 2011; Shimada et al., 2012). It is often unclear what determines the recognition of mtDNA by different PRRs leading to different immune responses. The same mitochondrial DAMPs can even trigger different PRRs in a tissue and cell-type specific manner (Rai et al., 2021).

Recent comprehensive reviews dissect the plethora of scenarios, in which mtDNA release has been observed and linked to mechanisms of immune activation (West and Shadel, 2017; Riley and Tait, 2020). Here, we explore how mitochondria provide a metabolic platform during innate immune signalling and how mtDNA release and innate immunity are coupled to metabolism. Firstly, we look at how mitochondria integrate metabolic regulation within effective innate immune responses to viral infection. We then go on to summarise the machinery and mechanisms that govern the release of mtDNA and discuss the emerging evidence that mtDNA localisation is controlled by cellular metabolism. Finally, we explore the metabolic outcomes of mtDNA release and touch upon the plethora of metabolic consequences of innate immune signalling.

## Mitochondrial Innate Immune Signalling During Infection

### MAVS Regulation by Glucose Metabolism

During viral infection, metabolic signals converge on the innate immune regulatory protein MAVS at the mitochondrial surface (Jacobs and Coyne, 2013). The multi-layered crosstalk between metabolism and MAVS at the OM drives innate immune responses to viral infection. The metabolic control of MAVS is dictated by direct metabolite and metabolic enzyme interactions and coupled to the dynamic behaviour of mitochondria.

**FIGURE 1:**
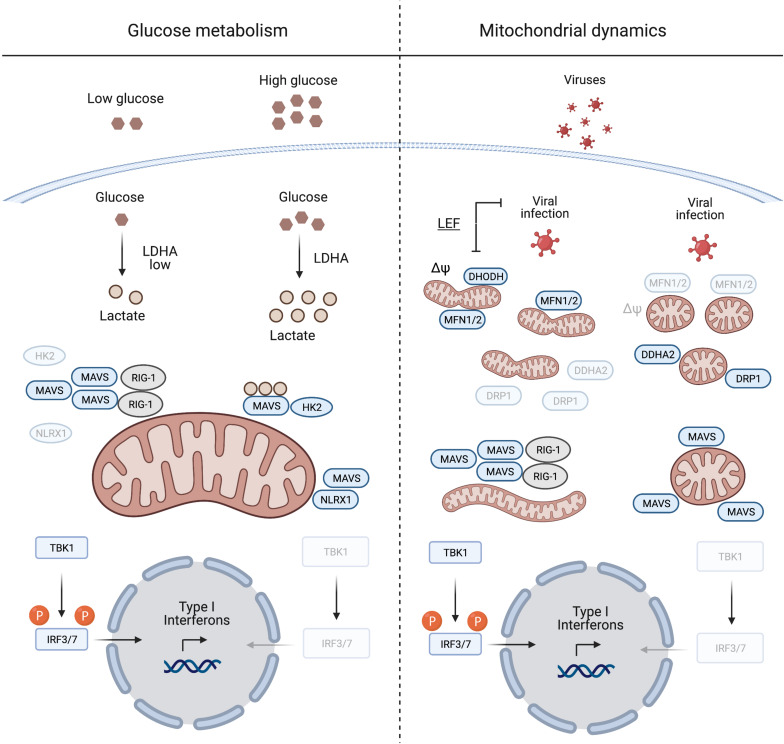
Mitochondrial antiviral signalling protein regulation induced by glucose metabolism and mitochondrial dynamics. During viral infection MAVS aggregation is essential for mounting an IFN response to suppress viral replication. Glucose and lactate levels (together with NLRX1) are negative regulators of MAVS aggregation (left panel). The right panel illustrates the pivotal role of mitochondrial dynamics in mounting an IFN response during viral infection. Inducing mitochondrial tubulation by expressing MFN2 or preventing DRP1 mitochondrial localisation mediated by DDHA2, as well as treating cells with the DHODH inhibitor leflunomide (LEF), supports MAVS aggregation and subsequent IFN response. Δψ, mitochondrial membrane potential. Created with BioRender.com.

The metabolic control of MAVS was described by Zhang et al. (2019), who identified lactate as a regulator of MAVS and RIG-I signalling. MAVS associates with the glycolytic enzyme hexokinase 2 (HK2) at the mitochondrial surface, where HK2 activity is regulated (John et al., 2011; Roberts and Miyamoto, 2015). Lactate binds to MAVS and prevents its aggregation, which is required for the activation of TBK1 and IFN production (Zhang et al., 2019; Figure 1). Limiting lactate production, for instance at low glucose levels or upon inhibition of lactate dehydrogenase A (LDHA), increases the IFN response to viral infection. Furthermore, MAVS activation leads to the dissociation and inactivation of HK2, which further decreases lactate levels (Zhang et al., 2019). Interestingly, similar to many cancer cells, virally infected cells shift their metabolism to aerobic glycolysis (DeBerardinis and Chandel, 2020; Zhou et al., 2021) and increase the production of lactate, which may be considered as an evolving mechanism employed by viruses to evade immune surveillance.

### NLRX1 and Mitochondrial Immune Signalling

Another innate immune regulatory protein that localises to mitochondria and is regulated by metabolic cues, is the nucleotide binding oligomerisation domain (NOD)-like receptor 1 (NLRX1). It negatively regulates the IFN response after viral infection via several mechanisms (Nagai-Singer et al., 2019; Pickering and Booty, 2021). During infection, NLRX1 interacts with MAVS to promote its ubiquitination and subsequent degradation (Qin et al., 2017), while the depletion of NLRX1 stabilises the MAVS-RIG-1 interaction and constitutively activates MAVS (Allen et al., 2011). Glucose depletion leads to decreased levels of NLRX1, which under these conditions will further unleash MAVS activation (Soares et al., 2014).

These studies thus further support the notion that the suppression of glucose metabolism activates MAVS. It should be noted, however, that NLRX1 inhibition of MAVS activity at the OM remains controversial. Studies performed in primary murine fibroblasts and mice lacking NLRX1 did not show any deficiency in elucidating an IFN response after viral infection (Rebsamen et al., 2011; Soares et al., 2013). Moreover, NLRX1 harbours a mitochondrial targetting sequence and was shown to translocate to the mitochondrial matrix (Arnoult et al., 2009).

### MAVS Signalling and Mitochondrial Dynamics

Mitochondrial antiviral signalling protein signalling is linked to mitochondrial fission and fusion, which drive the dynamic adaptation of the mitochondrial network to metabolic cues (Mishra and Chan, 2016; Wai and Langer, 2016). Mitochondrial fusion is controlled at the OM by the mitochondrial fusion proteins mitofusins 1 and 2 (MFN1/2), which regulate MAVS activity (Figure 1). Inhibition of fusion in response to viral infection decreases MAVS activity and IFN production (Koshiba et al., 2011; Pourcelot and Arnoult, 2014). On the other hand, increased mitochondrial fusion upon chemical inhibition of dihydroorotate dehydrogenase (DHODH), a mitochondrial inner membrane (IM) enzyme involved in *de novo* pyrimidine synthesis, prevents viral replication (Miret-Casals et al., 2018; Coelho and Oliveira, 2020; Xiong et al., 2020). Thus, mitochondrial fusion appears to be required for innate immune signalling. In line with this concept, the nitric oxide (NO) producer dimethylarginine dimethylaminohydrolase 2 (DDAH2) inhibits MAVS activity during viral infection by promoting mitochondrial fission (Huang et al., 2021). NO produced by DDAH2 in response to viral infection stimulates the phosphorylation of the dynamin-related protein-1 (DRP-1) and promotes mitochondrial fission. Cells lacking DDAH2 maintained an elongated mitochondrial network and MAVS-dependent IFN production after infection. Moreover, viral infection induces the expression of numerous miRNAs, amongst them miR-302b and miR-372, which target MAVS and limit the IFN response by promoting DRP-1 phosphorylation and mitochondrial fission (Yasukawa et al., 2020).

Together, these studies highlight the importance of a tubular mitochondrial network for mounting a MAVS dependent IFN response during infection. This agrees with the metabolic regulation of MAVS, since increased mitochondrial tubulation is usually associated with OXPHOS dependent cell growth, while glycolytic cells harbour a more fragmented mitochondrial network (Chen and Chan, 2017). Although it remains to be determined how mitochondrial dynamics and the shape of mitochondria affect immune signalling, an intricate interplay between antiviral signalling, mitochondrial dynamics and metabolism appears to exist. Further work will undoubtedly reveal other mechanisms by which MAVS translate metabolic changes initiated during viral infection into an effective IFN response. For the remainder of this review, we turn our attention to another metabolically sensitive regulator of innate immunity that normally resides in mitochondria: mtDNA.

## Metabolic Control of mtDNA-Dependent Innate Immunity

### The Release of mtDNA to the Cytosol

Mitochondria exhibit a pivotal role in mediating the IFN response upon infection by releasing mtDNA (or other DAMPs) to the cytosol, further boosting IFN production and immunosurveillance toward pathogens (Rongvaux et al., 2014; White et al., 2014; West et al., 2015; Sun et al., 2017). However, the presence of cytosolic or circulating mtDNA is also associated with chronic inflammation in a plethora of metabolic diseases and with ageing independent of bacterial or viral infection.

**FIGURE 2:**
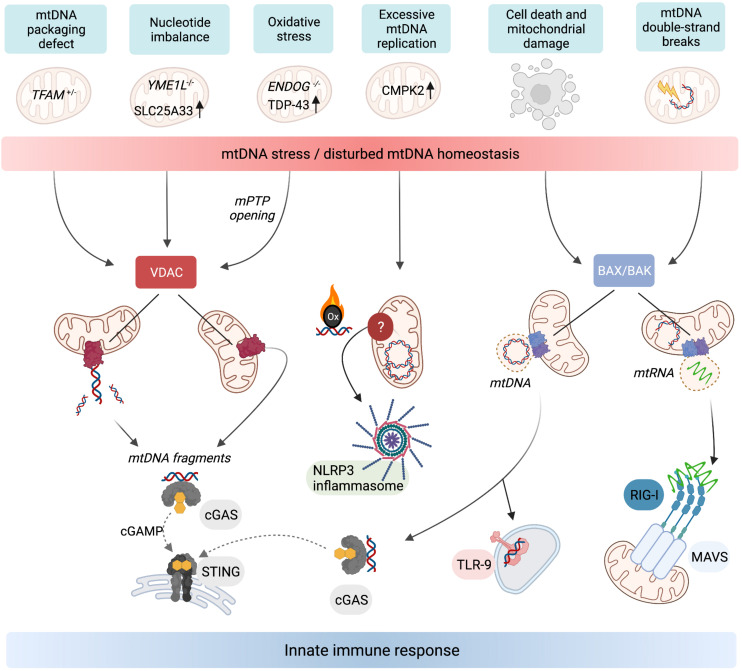
Release of mtDNA/RNA and the induction of innate immunity. Stressors, genetic, and metabolic perturbations triggering mtDNA/RNA-dependent innate immune responses. Fragments of mtDNA and mitochondrial nucleoids can be released along different pathways. VDAC oligomerisation at the OM allows the release of mtDNA fragments, which also requires concurrent opening of the mPTP in the mitochondria of *ENDOG*^–/–^ cells and neurons expressing mutant mitochondrial localised TDP-43. Oxidised mtDNA fragments are also released in macrophages undergoing excessive mtDNA replication and trigger the NLRP3 inflammasome. TFAM-bound mtDNA nucleoids are released from mitochondria in response to cell death signalling upon herniation of the IM through BAX/BAK pores in the OM. If the cell does not undergo apoptosis, the cytosolic mtDNA triggers innate immune signalling upon recognition by DNA-binding receptors including cGAS and TLR-9. BAX/BAK pores also facilitate the release of mtRNA from mitochondria that have been exposed to stressors which cause double-stranded breaks in mtDNA. Cytosolic mtRNA is recognised by RIG-I and triggers a RIG-I-MAVS-dependent innate immune response. Created with BioRender.com.

While the release of mtDNA from mitochondria is well established, the mechanisms allowing transfer to the cytosol are less clear. First insight was obtained by analysing the escape of mtDNA into the cytosol of apoptotic cells upon mitochondrial damage or various insults to tissue integrity and cellular homoeostasis. During apoptosis, mtDNA-protein assemblies termed nucleoids are released by herniation of the IM through large assemblies of BAX and BAK in the OM and subsequent IM permeabilisation (McArthur et al., 2018; Riley et al., 2018; Figure 2). mtDNA molecules are released under these conditions in association with the mitochondrial transcription factor A (TFAM), which serves as an mtDNA packaging factor. While this form of mtDNA release is predominantly immunologically silent owing to the activation of apoptotic caspases, a pro-inflammatory role for mtDNA release likely exists during the sub-lethal engagement of the mitochondrial apoptosis pathway, termed minority mitochondrial outer membrane permeabilisation (MOMP) (Ichim et al., 2015). Indeed, various pathogens were found to trigger minority MOMP and cytokine secretion from infected cells (Brokatzky et al., 2019).

Another form of mtDNA release occurs in non-apoptotic cells and does not require the formation of BAX/BAK pores but oligomerisation of VDAC in the OM instead (Figure 2). This mechanism, first described in cells lacking mitochondrial endonuclease G (ENDOG), involves the release of short mtDNA fragments (100–200 bp) rather than nucleoids and is also immunologically active, efficiently eliciting IFN response via cGAS-STING signalling (Kim et al., 2019). Chronic leakage of mtDNA through VDAC pores is associated with the induction of autoimmune diseases including Lupus (Kim et al., 2019) and amyotrophic lateral sclerosis (ALS) (Yu et al., 2020). The release of mtDNA fragments through VDAC pores in the OM is indeed emerging as a common pathway for the release of mtDNA to the cytosol, since treatment of cells with the VDAC oligomerisation inhibitor, VBIT4, is sufficient to blunt mtDNA-dependent innate immune responses in a number of contexts (Kim et al., 2019; Torres-Odio et al., 2020; Yu et al., 2020; Sprenger et al., 2021).

The mechanism(s) by which the IM is permeabilised to permit mtDNA export remain largely enigmatic. Opening the mitochondrial permeability transition pore (mPTP) can lead to mtDNA release (Patrushev et al., 2004; Garcia and Chavez, 2007). The pharmacological or genetic inactivation of mPTP is sufficient to block VDAC-dependent mtDNA release in *EndoG*^–/–^ cells and cells expressing a mutant form of TDP-43 associated with ALS (Kim et al., 2019; Yu et al., 2020). It remains to be seen whether mPTP opening is a pre-requisite for mtDNA release through VDAC pores in all cases and to what degree mPTP opening and VDAC oligomerisation are co-ordinated.

### The Release of mtDNA From Damaged Mitochondria

We are only beginning to understand what triggers the escape of mtDNA from mitochondria. mtDNA release during chronic inflammation in ageing and disease is mainly considered as the unwanted consequence of a decline in mitochondrial integrity. Mitophagy ensures the removal of damaged mitochondria and was found to counteract the release of mtDNA and the inflammatory response (Sliter et al., 2018). Mice lacking the pro-mitophagy protein Parkin, an E3 ubiquitin ligase mutated in familial cases of Parkinson’s disease, accumulate dysfunctional mitochondria and contain higher levels of circulating mtDNA following exhaustive exercise or genetic induction of mtDNA mutations (Sliter et al., 2018). Loss of Parkin drives inflammatory phenotypes under these conditions, which depend on STING. These data indicate that released mtDNA stimulates inflammation in these mice by activating the cGAS-STING pathway and highlights the immunosuppressive role of Parkin relevant to Parkinson’s disease (Matheoud et al., 2016; Sliter et al., 2018). Similar observations were made in tissues and cells derived from mice lacking the autophagy regulator protein immunity-related GTPase family M protein 1 (IRGM1) (Rai et al., 2021). In *Irgm1*^–/–^ fibroblasts, the release of mtDNA from dysfunctional mitochondria triggered an IFN response via cytosolic cGAS-STING, whereas the IFN response in *Irgm1*^–/–^ macrophages required the endosomal RNA sensor TLR7. This indicates an immunostimulatory role of extra-mitochondrial mtRNA in *Irgm1*^–/–^ macrophages, which may be another consequence of inefficient mitophagy and a decline in mitochondrial integrity (Rai et al., 2021).

### mtDNA Homoeostasis and Nucleotide Metabolism Control Innate Immunity

Many forms of metabolic perturbations compromise mitochondrial function and integrity, which lead to the cytosolic release of mtDNA (Figure 2). First evidence for the release of mtDNA independent of apparent gross mitochondrial dysfunction came from studies in cells heterozygous for the mitochondrial transcription factor and mtDNA packaging protein TFAM. TFAM-depleted cells exhibit mitochondrial stress linked to perturbed DNA packaging and constitutively release mtDNA from mitochondria to trigger cGAS-STING dependent ISG expression (West et al., 2015). It is unclear what stimulates the release of mis-packaged mtDNA and whether it is under metabolic control. Evidence of mtDNA oxidative stress upon TFAM depletion exists *in vivo* (Woo et al., 2012), which may signal its release or exacerbate the immunogenic potential of mtDNA (Caielli et al., 2016; Lai et al., 2018). The translation of *TFAM* mRNA is under control of the master metabolic regulator mTORC1 (Liu et al., 2017) but it remains to be seen whether TFAM levels or other factors affecting mtDNA stability or expression, such as the endonuclease ENDOG (Kim et al., 2019) or caseinolytic mitochondrial matrix peptidase subunit CLPP (Torres-Odio et al., 2020), are directly regulated by metabolic pathways to promote or repress mtDNA release.

An inflammatory response is also mounted against the accumulation of errors during mtDNA replication. Mitochondrial polymerase gamma (POLG) mutator mice harbour excessive mtDNA mutations due to insufficient proof-reading and exhibit pathologies common in human mitochondrial diseases and premature ageing (Trifunovic et al., 2004; Kujoth et al., 2005). New results indicate that mutator mice exhibit enhanced activation of the cGAS-STING pathway associated with mtDNA release after LPS challenge and innate immune stimuli (Lei et al., 2021) or in the absence of mitophagy (Sliter et al., 2018). Blocking the hyperactive type I IFN response alleviates the pro-inflammatory metabolic phenotypes of mutator mice, including oxidative stress and aerobic glycolysis, and extends lifespan (Lei et al., 2021). Tigano et al. (2021) recently discovered that excessive mtDNA breaks can cause BAX/BAK-dependent release of mtRNA to activate a RIG-I-MAVS-dependent immune response. The cytosolic release of mtRNA has also been observed upon an excessive accumulation of double-stranded RNA in mitochondria (Dhir et al., 2018). Further work is necessary to understand how mtDNA integrity or replication rates are sensed and translated into immune responses downstream of different mitochondrial DAMPs.

Mitochondrial genome maintenance is coupled to cellular nucleotide metabolism and homoeostasis (El-Hattab et al., 2017). Recent work revealed that nucleotide synthesis and balance impacts mtDNA stability and regulates its release into the cytosol (Zhong et al., 2018; Sprenger et al., 2021). Macrophage priming with LPS causes the cytosolic exposure of mtDNA and subsequent activation of the NLRP3 inflammasome (Nakahira et al., 2011; Zhong et al., 2018). LPS-primed macrophages undergo a surge in mtDNA synthesis upon transcriptional upregulation of the mitochondrial deoxyribonucleotide kinase CMPK2 (Zhong et al., 2018). CMPK2 phosphorylates dCMP to dCDP in the mitochondrial deoxynucleoside triphosphate (dNTP) salvage pathway and is rate limiting for the accumulation of newly synthesised oxidised mtDNA in primed macrophages, which stimulates the inflammasome upon exposure to the cytosol (Zhong et al., 2018; Figure 2). These findings demonstrate that mitochondrial nucleotide metabolism is coupled to the replication and release of mtDNA.

Nucleotides are synthesised *de novo* from amino acids and ribose precursors in the cytosol or assembled via the mitochondrial dNTP salvage pathway from free nucleosides and bases. Increasing evidence suggests that nucleotide uptake into mitochondria limits the accumulation of mtDNA in proliferating cells (Favre et al., 2010; Di Noia et al., 2014; Sprenger et al., 2021). We recently observed that disturbances in cellular nucleotide homoeostasis, including deregulated nucleotide uptake, triggers the release of mtDNA (Sprenger et al., 2021). Mouse retina and cultured cells lacking the mitochondrial protease YME1L exhibit a specific innate immune response that, in cultured cells, depends on mtDNA-dependent cGAS-STING activation (Sprenger et al., 2021). YME1L is a multifaceted metabolic regulator in the IM that programmes the mitochondrial proteome to maintain pyrimidine nucleotide synthesis via glutaminolysis and to modulate the transport of pyrimidines across the IM via proteolysis of the pyrimidine nucleotide carrier SLC25A33 (MacVicar et al., 2019; Sprenger et al., 2021). Loss of YME1L results in the accumulation of SLC25A33 and cellular depletion of pyrimidine nucleotides, which triggers the release of mtDNA into the cytosol (Figure 2). mtDNA release occurs in a BAX/BAK-independent manner but depends on VDAC oligomerisation in the OM, which also governs the release of mtDNA fragments in cells lacking ENDOG (Kim et al., 2019). Both accumulation of SLC25A33 or inhibition of pyrimidine synthesis independently are sufficient to induce mtDNA release from mitochondria. Depletion of cellular pyrimidines via genetic intervention or treatment with the pyrimidine nucleoside analogue 5-fluorouracil (5-FU) triggered mtDNA-dependent cGAS-STING activation, demonstrating that this mtDNA release pathway may be of relevance in a number of clinical contexts (Sprenger et al., 2021). For instance, cGAS-STING dependent type I IFN production is triggered by 5-FU in cancer cells and supports effective anti-tumour immunity in colorectal cancer (Tian et al., 2021). Intriguingly, *YME1L* was found to be frequently mutated in colorectal cancer tissue, which may drive the migration of mtDNA to the nucleus observed in these cancers (Srinivasainagendra et al., 2017).

It is exciting to consider that pyrimidine depletion is a common trigger of mtDNA release and innate immunity. Cytosolic exposure of mtDNA contributes to cellular antiviral responses since enhanced expression of the specific panel of ISGs downstream of mtDNA-dependent cGAS-STING signalling supports antiviral immunity (West et al., 2015). Of note, pyrimidine analogues are employed as antiviral agents and the fact that they induce type I IFN responses has been appreciated for some time (Lucas-Hourani et al., 2013; Shin et al., 2018). It will be important to ascertain whether mtDNA exposure supports innate immune responses to nucleoside analogues in antiviral and anti-tumour signalling. It will also be pertinent to establish the degree to which mtDNA release synergises with nuclear DNA damage responses to nucleotide depletion (Hastak et al., 2008; Hamalainen et al., 2019). Interestingly, some viral infections such as Dengue virus induce innate immune responses associated with mtDNA stress and release (Sun et al., 2017; Lai et al., 2018) and mtDNA has been detected in the blood of hospitalised COVID-19 patients (Scozzi et al., 2021). While the presence of circulating mtDNA in virus infected individuals likely results mainly from tissue damage and cell death, it remains to be seen whether infected cells also sense nucleotide imbalance during viral infection and respond by releasing mtDNA to drive innate immunity.

### Disturbed Lipid Homoeostasis and mtDNA Release

In addition to nucleotides, the metabolic homoeostasis of other macromolecules, especially lipids, has also been linked to mtDNA-dependent innate immunity. Disturbances in the synthesis of lipids in adipose tissue impair mitochondrial respiration and enhance ROS production (Bai et al., 2017). This is associated with the release of mtDNA and the induction of cGAS-STING-dependent inflammation and insulin resistance during obesity (Bai et al., 2017). Lipid metabolism also regulates mtDNA release in immune cells. Regulatory T cells (Tregs) suppress immune responses by secreting cytokines such as IL-10 to arrest effector T cell proliferation (Chaudhry et al., 2011). The suppressive capability of Tregs is enhanced upon acute inhibition of the fatty acid binding protein FABP5, which was associated with a decline in fatty acid oxidation (Field et al., 2020). Pharmacological inhibition of FABP5 triggered the release of mtDNA and stimulated a type I IFN response in a cGAS-STING dependent manner. A similar innate immune response was engaged in Tregs within solid tumours indicating that mtDNA-dependent signalling may occur upon exposure to a lipid-depleted microenvironment (Field et al., 2020). While the presence of cytosolic mtDNA in Tregs correlated with a decline in OXPHOS and disturbed mitochondrial cristae morphology, it is unclear how mtDNA is released from these mitochondria especially since limiting OXPHOS and disturbing cristae morphogenesis in these cells by other means does not trigger mtDNA release (Field et al., 2020). Finally, metabolic dysregulation in renal tubule cells that rely heavily on fatty acid oxidation is associated with transcriptional repression of the mtDNA binding protein TFAM and mtDNA-dependent cGAS-STING activation in kidney disease and fibrosis (Chung et al., 2019), further indicating a link between disturbed fatty acid metabolism and mtDNA release.

## Metabolic Consequences of mtDNA Release and Innate Immune Signalling

**FIGURE 3:**
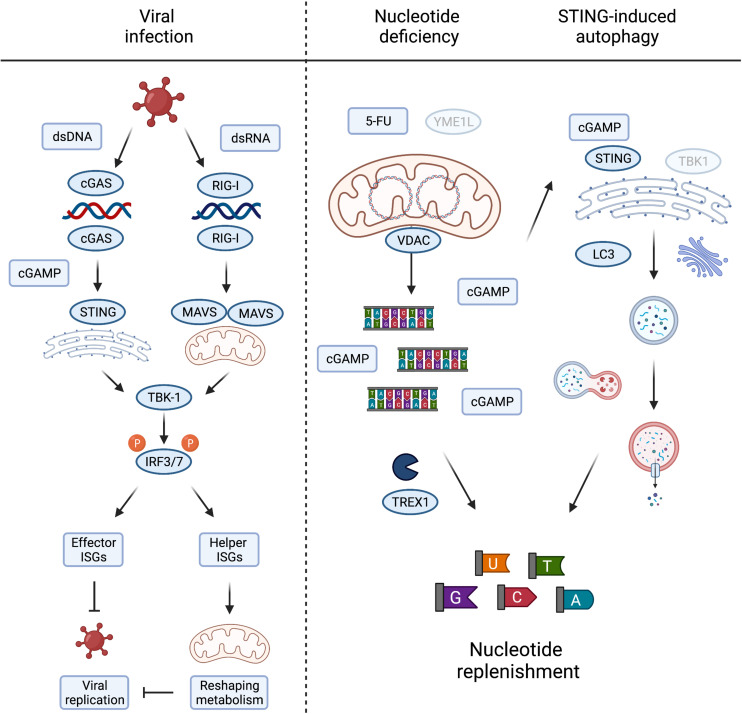
Metabolic consequences of mtDNA release and innate immune signaling. Viral infection, either by DNA or RNA viruses, leads to the activation of TBK1 and subsequent phosphorylation of IRF3/7 to induce their nuclear translocation to activate type I interferons. The interferon stimulated genes (ISGs) prevent viral growth, either by direct inhibition of viral replication, termed here “effector ISGs,” or indirectly by rewiring mitochondrial and cellular metabolism (“helper ISGs”). The latter could be achieved by inducing metabolic conditions supporting/favouring the release of mtDNA **(left panel)**. Nucleotide deficiency induced by loss of YME1L or treatment with thymidylate synthase (TS) inhibitor 5-FU leads to the release of mtDNA. TREX1 competes with cGAS for the binding and degradation of mitochondrial dsDNA to single nucleotides, replenishing cellular nucleotide pools in the process. cGAS binding of mtDNA generates cGAMP to activate STING-induced autophagy, independent of TBK1 activation, and innate immunity, by LC3 lipidation and autophagosome formation. The autophagy/lysosome system further contribute to the replenishment of cytosolic nucleotide pools **(right panel)**. Created with BioRender.com.

Compelling evidence demonstrates that metabolic signals direct mitochondria-dependent immune responses and ISG expression, which restrict viral replication. However, only a subset of ISGs limits viral replication directly and the antiviral function for the majority of ISGs is unknown. Many ISGs reshape cellular and mitochondrial metabolism to activate immune cells and suppress infection indirectly (Figure 3). For instance, reprogramming host cell nucleotide metabolism in response to infection can limit the pool of dNTPs available for viral DNA replication (Lahouassa et al., 2012) and IFN signalling rewires oxidative metabolism and alters citric acid cycle metabolite levels to facilitate the reprogramming of dendritic cells and macrophages (O’Neill and Pearce, 2016; Timblin et al., 2021). The reciprocal relationship between innate immune response and metabolism is also illustrated by the metabolic phenotypes associated with chronic immune activation in disease (Fritsch and Weichhart, 2016; Raniga and Liang, 2018). While many intriguing aspects of immunometabolism lie beyond the scope of this review, understanding more about the function(s) of individual ISGs specifically expressed during mtDNA-dependent innate immunity will help us to decipher the metabolic consequences of mtDNA release and its role in disease.

It is fascinating to consider that mtDNA release to the cytosol during sterile inflammation serves additional metabolic purposes besides engaging innate immune signalling pathways. The loss of the evolutionary conserved mitochondrial protease YME1L induces mtDNA release and cGAS-STING signalling in mouse and human cells. The stimulation of mtDNA release in YME1L deficient cells inherently devoid of inflammation signalling pathways, such as *yme1* mutant yeast, argues that extra-mitochondrial mtDNA could have alternative conserved roles to play (Thorsness et al., 1993). This includes the migration of mitochondrial genetic material to the nucleus (Thorsness and Fox, 1990; Thorsness et al., 1993) but may also fulfil other signalling duties when triggered by nucleotide depletion. It is another intriguing possibility that mtDNA also serves as a storage for nucleotides that can be released into the cytosol under conditions when cytosolic nucleotide pools are limiting (Sprenger et al., 2021). Consistently, depletion of the cytosolic three prime repair exonuclease 1 (TREX1) boosted cGAS-dependent immune signalling in *YME1L*^–/–^ cells suggesting competition between degradation of cytosolic mtDNA and cGAS binding (Sprenger et al., 2021).

Pools of nucleotides and their metabolic precursors can also be maintained via the autophagy pathway and lysosomal recycling (Guo et al., 2016; Liu et al., 2018). Pyrimidine and purine depletion can induce autophagy via different pathways (Hoxhaj et al., 2017; Mimura et al., 2021) that may synergise with the release of mtDNA to replenish nucleotide pools. Gui et al. (2019) recently described the induction of autophagy as a primordial function of dsDNA recognition by cGAS-STING, whereby cGAMP triggers STING translocation to the ER-Golgi intermediate compartment to trigger autophagosome formation independent of IFN production. Delineation and functional analysis of the autophagosome-inducing STING domain revealed that the autophagy activity exists in ancient STING homologues, which are otherwise incapable of activating IFNs via TBK1 (Kranzusch et al., 2015; Gui et al., 2019). While autophagy induction by STING does not appear to be sufficient to drive an effective STING-mediated antiviral response in mice, which rather depends on STING-TBK1 signalling (Yum et al., 2021), autophagy can help defend cells against pathogens by delivering them to the lysosome for degradation and supports the clearance of cytosolic DNA (Gui et al., 2019). It is therefore tempting to speculate that mtDNA release supports autophagy induction via cGAS-STING signalling in a further attempt to restore depleted nucleotide levels (Figure 3). In line with this possibility, yeast autophagy mutants under starvation conditions were shown to degrade mtDNA, perhaps further releasing it to replenish cytosolic nucleotides otherwise limiting under these conditions (Medeiros et al., 2018). Further work will be required to investigate the contribution of cytosolic mtDNA to cellular nucleotide metabolism, which will bear significance in scenarios where nucleotide balance is disturbed, including tumour cell responses to chemotherapy and host cell responses to pathogen infection.

## Concluding Remarks

Mitochondria play essential roles at the crossroads of metabolism and innate immunity. The dynamic mitochondrial network integrates MAVS signalling with glucose metabolism during viral infection and releases mtDNA to drive inflammatory responses to distinct metabolic signals. mtDNA release is frequently observed in pathological scenarios and evidence is accumulating that metabolic signals can induce it. On the other hand, a sentinel role played by cytosolic mtDNA in metabolism and innate immune signalling is emerging.

Many important questions await to be answered, including how these metabolic signals are relayed to the mitochondria in order to expel mtDNA and what determines whether mtDNA is released in fragments via VDAC or as whole nucleoids via BAX/BAK pores. It will be fascinating to address whether metabolic signalling programmes control the selective packaging and extracellular extrusion of mtDNA via exosomes. Recently, Todkar et al. (2021) reported that mitochondrial derived vesicles (Sugiura et al., 2014) control the incorporation of mitochondrial proteins and mtDNA into exosomes in cultured cells. Finally, what is the physiological role of regulated mtDNA release and can it be manipulated, for example in order to improve chemotherapy or treat autoimmune diseases? It has become clear that cytosolic mtDNA can have profound effects on cellular metabolism and it will be exciting to see if targetting these metabolic pathways prove beneficial in therapy.

## Author Contributions

All authors listed have made a substantial, direct and intellectual contribution to the work, and approved it for publication.

## Conflict of Interest

The authors declare that the research was conducted in the absence of any commercial or financial relationships that could be construed as a potential conflict of interest.

## Publisher’s Note

All claims expressed in this article are solely those of the authors and do not necessarily represent those of their affiliated organizations, or those of the publisher, the editors and the reviewers. Any product that may be evaluated in this article, or claim that may be made by its manufacturer, is not guaranteed or endorsed by the publisher.
